# Impact of a modified data capture period on Liu comorbidity index scores in Medicare enrollees initiating chronic dialysis

**DOI:** 10.1186/1471-2369-14-51

**Published:** 2013-02-27

**Authors:** Sally K Rigler, James B Wetmore, Jonathan D Mahnken, Lei Dong, Edward F Ellerbeck, Theresa I Shireman

**Affiliations:** 1 Department of General and Geriatric Medicine, University of Kansas School of Medicine, 3901 Rainbow Boulevard, MS 1037, Kansas City, KS, 66160, USA; 2 Department of Nephrology and Hypertension, University of Kansas School of Medicine, 3901 Rainbow Boulevard, MS 3002, Kansas City, KS, 66160, USA; 3 Department of Biostatistics, University of Kansas School of Medicine, 3901 Rainbow Boulevard, MS 1026, Kansas City, KS, 66160, USA; 4 Department of Preventive Medicine and Public Health, University of Kansas School of Medicine, 3901 Rainbow Boulevard, MS 1008, Kansas City, KS, 66160, USA; 5 Office of Scholarly, Academic & Research Mentoring (OSARM), University of Kansas Medical Center, 3901 Rainbow Blvd., Mail Stop 1037, Kansas City, KS, 66160, USA

**Keywords:** Comorbidity, Kidney failure, Chronic, Renal dialysis, Epidemiologic research design

## Abstract

**Background:**

The Liu Comorbidity Index uses the United States Renal Data System (USRDS) to quantify comorbidity in chronic dialysis patients, capturing baseline comorbidities from days 91 through 270 after dialysis initiation. The 270 day survival requirement results in sample size reductions and potential survivor bias. An earlier and shorter time-frame for data capture could be beneficial, if sufficiently similar comorbidity information could be ascertained.

**Methods:**

USRDS data were used in a retrospective observational study of 70,114 Medicare- and Medicaid-eligible persons who initiated chronic dialysis during the years 2000–2005. The Liu index was modified by changing the baseline comorbidity capture period to days 1–90 after dialysis initiation for persons continuously enrolled in Medicare. The scores resulting from the original and the modified comorbidity indices were compared, and the impact on sample size was calculated.

**Results:**

The original Liu comorbidity index could be calculated for 75% of the sample, but the remaining 25% did not survive to 270 days. Among 52,937 individuals for whom both scores could be calculated, the mean scores for the original and the modified index were 7.4 ± 4.0 and 6.4 ± 3.6 points, respectively, on a 24-point scale. The most commonly calculated difference between scores was zero, occurring in 44% of patients. Greater comorbidity was found in those who died before 270 days.

**Conclusions:**

A modified version of the Liu comorbidity index captures the majority of comorbidity in persons who are Medicare-enrolled at the time of chronic dialysis initiation. This modification reduces sample size losses and facilitates inclusion of a sicker portion of the population in whom early mortality is common.

## Background

Studies using large, observational data sets are vitally important sources of evidence for clinical populations in which randomized trials are difficult to conduct [1-3]. Persons with advanced renal disease are excluded from most randomized, clinical trials (RCT) of treatments for other disease states [4-8]. Even when feasible, RCT results may not generalize well to broader clinical populations because only the most select subset of persons can be enrolled and randomized. Observational studies improve generalizability but are fraught with treatment selection bias [9]. Valid measures of comorbid disease burden and mortality risk must therefore be incorporated to adjust for non-random treatment selection in real-world care [10,11].

Chronic dialysis patients have higher rates of cardiovascular comorbidity and reduced survival in comparison to the general population from which most comorbidity indices are derived [12,13]. Thus, comorbidity measures designed specifically for the chronic dialysis population may be preferable [14,15]. The United States Renal Data System (USRDS) is an important source of observational data for studies of the chronic dialysis population [16]. The USRDS incorporates demographic information, Medicare claims (permitting claims-and procedure-based diagnoses to be captured), and baseline health status information from the CMS Medical Evidence Form (CMS 2728) which is completed at the time of dialysis initiation.

In 2010, Liu et al. created a new comorbidity index using USRDS data as a basis [17]. Because new chronic dialysis initiates may take up to 90 days to acquire Medicare eligibility, claims data were frequently found to be missing during the first 90 days after dialysis initiation. Thus, these investigators set the start-date for capturing Medicare claims-based diagnoses at day 91. A subsequent six month period for comorbidity capture was used, spanning days 91 to 270 after dialysis initiation. Outcomes were then evaluated from day 271 onward. This index performed better than the traditional Charlson Comorbidity Index (CCI) and was predictive of mortality, hospitalization and medical costs [17].

The current study is prompted by concerns about the time period required to acquire claims-based diagnoses codes for calculation of the original Liu comorbidity score. If the original Liu index can only be applied to patients who survive at least 270 days after starting dialysis, then it limits the study sample to a healthier subset of dialysis initiates. Because more than one in five patients starting dialysis will not survive their first year [17], such a survival requirement will reduce sample size and lessen the generalizability of study results because sicker patients are excluded from study [18]. The alternative is for investigators to apply other comorbidity indices which were not developed specifically for this unique population, and neither option is ideal.

To meet study purposes for our own USRDS-based projects, our research sample includes only persons who are already Medicare-eligible at the time of dialysis initiation. Because Medicare claims data are thus available in the first 90 days for all subjects, we explored the option of commencing the capture of baseline comorbidity immediately upon dialysis initiation. In addition, we reasoned that a shorter time period for baseline comorbidity ascertainment would also help reduce sample size losses and improve generalizability, while allowing the post-baseline outcomes assessment period to begin sooner. However, we recognized that a shorter baseline period for observing Medicare claims-based diagnoses might impede total comorbidity ascertainment. The results of “trading off” time in the baseline period (where baseline comorbidities are captured) for time in the post-baseline period (where outcomes are captured) have not been studied with this index. In chronic dialysis, where median survival is less than three years after dialysis initiation, this is a pressing design issue for observational studies [16,17].

Thus, our goals were to examine the impact of modifying the Liu Comorbidity Index by: (a) shifting the start date for comorbidity capture forward to begin immediately after dialysis initiation (Day 0), and (b) shortening the total baseline period for comorbidity capture from 180 days to 90 days. We applied these changes to a population with Medicare coverage in place at the time of dialysis initiation. We characterized the impact on sample size and compared the comorbidity scores resulting from the original index and the new modification.

## Methods

### Sample

This study arose from a larger project in which USRDS data was linked to Medicaid pharmacy claims for the period 1/1/2000-12/31/2005. All subjects were dually-eligible for both Medicare and Medicaid from the time of dialysis initiation and throughout subsequent observation. Subjects with managed care plans and those receiving care in the Veterans Affairs system were excluded because their health care claims could not be observed. Other pragmatic data limitations required exclusion of residents of Arizona, Tennessee and the United State Territories. Persons who moved from one state to another during observation were also excluded.

All subjects had to survive the first 90 days or more after dialysis initiation without receiving kidney transplantation. From this larger pool of chronic dialysis patients, an incident cohort of persons initiating dialysis was selected. The derivation of this incident cohort has been previously reported elsewhere [19].

### Variables

The CMS 2728 form provided data from the time of dialysis initiation, including sociodemographic factors (age, gender, race/ethnicity, employment, tobacco use, substance abuse), general health status factors (inability to ambulate, inability to transfer, body mass index), and renal health factors (primary cause of end stage renal disease, in-center versus home dialysis, hemoglobin adequacy indicator). Selected comorbid disease (e.g., diabetes mellitus, heart failure, coronary artery disease, cerebrovascular disease, peripheral vascular disease) also came from the CMS 2728 form. The remaining conditions of interest were captured from Medicare claims using standard methods for inferring the presence of disease from service claims linked to ICD-9-CM codes for these conditions [17].

As per the original Liu index, diagnoses were considered present if they were found on either the CMS 2728 or in Medicare claims.

### Two versions of the Liu Comorbidity Index

We first calculated the original Liu Comorbidity Index, gathering Medicare claims-based diagnoses from days 91 to 270 after dialysis initiation to supplement conditions recorded on the CMS 2728. Briefly, this index assigns 11 conditions the following weights: 1 point for atherosclerotic heart disease and for diabetes; 2 points for cerebrovascular accident/transient ischemic attack, peripheral vascular disease, dysrhythmia, other cardiac disease, chronic obstructive pulmonary disease, gastrointestinal bleeding, liver disease and cancer; and 3 points for congestive heart failure. We used the version of the original Liu index which also assigns points for the primary cause of ESRD, as follows: glomerulonephritis (0 point), hypertension (2 points), diabetes (3 points), and other causes (3 points). In this form, the Liu Comorbidity Index results in a score ranging from 0 to 24 points [17].

Next, we modified the original Liu index. We retained the same variables and scoring weights but with two modifications to the time-frame of baseline comorbidity capture. First, we allowed claims-based diagnoses capture to commence immediately upon dialysis initiation, instead of forcing a 90-day waiting period. Second, we reduced the total baseline claims capture period from a 180 day total span (days 91–270 in the original version) to a 90 day span (days 1–90 in the modified version). This newly modified comorbidity index is henceforth called the Modified Liu Index _1–90_, to indicate the days in which claims are captured.

### Analysis

Demographic and clinical characteristics were derived for the overall cohort and for the relevant comorbidity index subgroups, as follows. We calculated the proportion of our overall cohort who survived long enough to be eligible to have been included in both comorbidity indices; i.e., at least 270 days of survival after dialysis initiation for the original Liu Index and at least 90 days for the Modified Liu Index _1–90_. We calculated the Modified Liu Index _1–90_ for all subjects, and we also calculated the original Liu Index on the sample subgroup who would have been eligible for both. The numeric score difference between these two indices was calculated for each subject who had both measures calculated. Descriptive statistics were obtained for the score differences. Bubble plots were examined to assess overlap between the two measures and the Pearson correlation coefficient was estimated, along with 95% confidence intervals. To further evaluate the Modified Liu Index _1–90_ as an estimate of the original Liu Index, we conducted a one-sample t-test of the score difference to test for bias (indicated if the average difference between the two measures was not zero) in this estimate and calculated the mean square error (MSE) [20]. The MSE was estimated by the sum of the sample variance for the original Liu Index and the square of the bias. The proportions of subjects whose differences were zero, within one, two, three, four, and five were estimated along with their corresponding 95% Wald confidence intervals. A histogram of the score differences was also generated with a normal distribution overlaid.

To evaluate differences in demographic and clinical characteristics between those for whom both comorbidity measures were calculated (i.e., those that survived at least 270 days on dialysis) versus those that survived at least 90 days (but less than 270 days), bivariate analyses were conducted. This included contingency tables and the Pearson chi-square test for categorical measures, and means, standard deviations, and the two-sample t-test for continuous measures.

SAS version 9.2 (SAS Institute, Inc., 2002–2008) was used for data management and analysis. This study was approved by the University of Kansas Medical Center’s Human Subjects Committee. Data were used under data use agreements with the USRDS and the Centers for Medicare & Medicaid Services.

## Results

There were 70,114 subjects with complete data. The original Liu Index could be calculated for 75% of these (n = 52,937), while the remaining 25% (n = 70,114-52,937) did not survive long enough to meet the requirements for calculation of the original Liu Index. Table 1 shows the demographic and clinical characteristics of the overall cohort and these two subgroups.

**Table 1:** Demographic & clinical characteristics of study sample

**Variable**	**Total cohort (n = 70,114)**	**Survived < 270 days (n = 17,177)**	**Survived ≥ 270 days (n = 52,937)**	^**± **^**p-value**
Comorbidity index scores				
*Original Liu Comorbidity Index Score (mean ± st. dev.)	Cannot be calculated	Cannot be calculated	7.35 ± 3.99	N/A
**Modified Liu Comorbidity Index Score (mean ± st. dev.)	6.60 ± 3.75	7.30 ± 4.16	6.37 ± 3.58	<0.0001
Demographic and clinical characteristics				
Age (years) (mean ± st. dev.)	61.5 ± 15.4	62.3 ± 16.4	61.2 ± 15.1	<0.0001
Gender (% women)	56.2%	53.8%	57.0%	<0.0001
Race/Ethnicity				<0.0001
Black	40.3%	39.0%	40.8%	
Hispanic	18.0%	15.8%	18.8%	
Other	6.0%	5.2%	6.2%	
White	35.7%	40.0%	34.3%	
Employed	2.5%	3.2%	2.3%	<0.0001
Tobacco use	6.4%	6.4%	6.4%	0.7830
Substance abuse	2.9%	2.9%	2.8%	0.7413
Unable to ambulate	5.8%	7.9%	5.2%	<0.0001
Unable to transfer	2.1%	3.3%	1.8%	<0.0001
Home dialysis	4.9%	4.6%	5.0%	0.0589
Body Mass Index				<0.0001
<20	10.4%	12.5%	9.7%	
≥20 to < 24.99	30.0%	31.5%	29.5%	
≥25 to <29.99	26.9%	26.2%	27.1%	
≥30	32.8%	29.9%	33.7%	
Hemoglobin >11 mg/dL	25.0%	24.8%	25.0%	0.4501
Primary renal diagnosis:				<0.0001
Diabetes mellitus	53.0%	49.3%	54.2%	
Hypertension	25.7%	26.5%	25.4%	
Immune	7.4%	7.5%	7.3%	
Other	14.0%	16.8%	13.1%	
¶Diabetes mellitus	62.8%	60.3%	63.7%	<0.0001
Congestive heart failure	34.2%	37.1%	33.3%	<0.0001
Coronary artery disease	25.4%	27.2%	24.8%	<0.0001
Cerebrovascular disease	11.1%	12.5%	10.6%	<0.0001
Peripheral vascular disease	14.8%	16.6%	14.3%	<0.001

*Original Liu Index captures comorbidity data from days 91–270 after dialysis initiation.

** Modified Liu Index _1–90_ captures comorbidity data from days 1–90 after dialysis initiation.

± p-value for bivariate test statistic by survival group.

¶ Diabetes considered present if either noted as the primary cause for renal failure or as a comorbidity in claims data.

**Figure 1:**
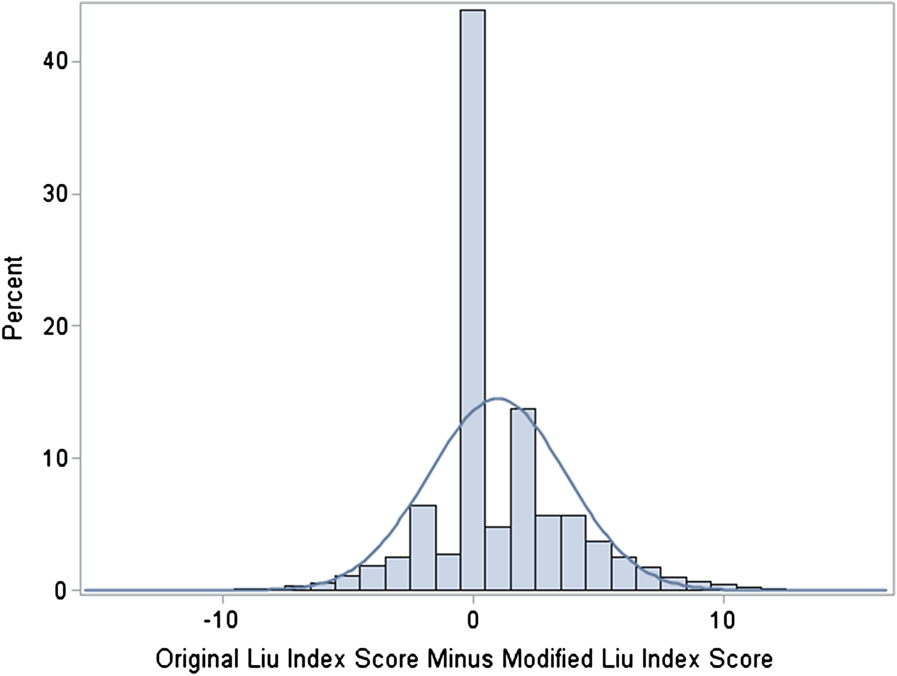
Distribution of difference between original and modified Liu index scores.

For subjects who survived long enough to have both versions of the index calculated, good overlap between the two comorbidity scores was observed using bubble plots (not shown); the Pearson correlation coefficient was 0.74 (95% confidence interval: 0.73-0.74). The median and mode for the difference between the comorbidity scores were both equal to zero. See Figure 1. Mean scores ± standard deviation for the original index and the modified index were 7.4 ± 4.0 and 6.4 ± 3.6 points, respectively, generating a difference (estimated bias) of 0.98 ± 2.8 points (p < 0.0001 for test of no bias). The MSE for the Modified Liu Index _1–90_ as an estimator of the original Liu Index was approximately 16.87, making the root MSE 4.1. The two scores were exactly equal for 43.9% (95% confidence interval: 43.5-44.3%) of individuals. The proportions with differences within one to five points were (respectively) 51.4%, 71.6%, 79.7%, 87.3%, and 92.1%, with each having narrow confidence intervals similar to that for those whose scores were exactly equal.

For the 25% of subjects who lived at least 90 days but less than 270 days, the mean Modified Liu Index _1–90_ score was 7.3 ± 4.2 points. This group differed in health status from those who survived the 270 days on most variables, as shown in Table 1, but the differences between groups were generally not more than a few percentage points.

## Discussion

Our goals were to determine the impact of a modified data capture period on Liu Comorbidity Index values for a large sample of incident chronic dialysis patients in whom Medicare coverage was already in place at the time of dialysis initiation. We found that approximately 32% more subjects could be retained if the comorbidity capture period was modified to require survival to only the first 90 days after dialysis initiation. These shorter survivors were, as expected, somewhat sicker on average than those who survived into the 91–270 day capture period (or longer), as evidenced by a slightly higher comorbidity score (approximately one point higher on a 24 point scale).

The beneficial effects of allowing 0 –90 day comorbidity capture were to enhance the study size and to expand the breadth of baseline health status in the subject pool. For study design purposes, this shorter baseline health ascertainment period also has the beneficial impact of allowing for a longer period of post-baseline follow-up observation in which subjects experience the health outcomes of interest. This may be an especially attractive feature for observational studies populations with shortened survival, such as is the case with chronic dialysis patients.

Conversely we were concerned that the shorter period of claims-based comorbidity ascertainment could result in reduced ascertainment of overall comorbidity because fewer health care claims would be generated during the shorter observation period. A modest difference was indeed noted, but it averaged only a single point on a 24-point scale. Indeed, the most common change in comorbidity score was no change at all, present in nearly half the individuals. For other investigators, we would suggest that this modest reduction in ascertainment of comorbidity (which is typically used as one of many adjustment covariates) is a price well worth paying in order to prevent a one-third sample size loss and the elimination of the sickest patients from a study cohort.

### Existing comorbidity measures

The Liu index is important because (a) it was developed specifically for use in the chronic dialysis population; and (b) it relies only upon diagnoses derived from the CMS 2728 form and/or Medicare claims. No additional clinical measures are needed, making it practical for application to a host of observational claims-based studies in the USRDS. However, it must be noted that other investigators have used pre-existing comorbidity tools to successfully characterize the impact of comorbidity on outcomes in chronic dialysis populations. Published literature generally supports the validity of these general comorbidity measures in this population, despite the fact that they were developed in dissimilar clinical populations.

However, most of these studies have relied upon first-hand clinical data rather than administrative claims, so their applicability to administrative claims-based studies is unclear. Hemmelgarn et al. adapted the Charlson comorbidity index [21] for use in chronic dialysis patients, finding only a slight advantage for the modified version [22]. However, comorbid diagnoses were abstracted from clinical chart data. The studies of van Manen et al. showed three existing comorbidity indices (Khan, Davies and Charlson) to be appropriate for characterizing the impact of comorbidity on mortality [23] and on health status and quality of life in chronic dialysis patients [24]. However, in this case, data on the presence and severity of comorbid diagnoses came from each patient’s nephrologist.

Other investigators have developed tools specifically for the chronic dialysis population but which require data sources that are not available in claims. Miskulin et al. used the Index of Coexistent Disease to characterize comorbid disease burden at the time of dialysis initiation and thereafter, finding that the baseline ICED score was the best independent predictor of mortality during subsequent follow-up [25]. However, along with disease severity indicators, this index also incorporates physical impairment information that is not available from the USRDS.

### Limitations

Study limitations must be noted. First, this study sample was comprised only of persons who were dually eligible for both Medicare and Medicaid; results may not be generalizable to other chronic dialysis patients [26]. Second, these results apply only to persons who are already Medicare eligible at the time of dialysis initiation. However, this is a large subset of the chronic dialysis population; 58% of new hemodialysis patients have non-managed care Medicare coverage [16]. Third, as with any claims-based comorbidity index, diseases are ascertained only if there are coded health care service claims available in the administrative data sets; these data were not confirmed with direct evidence from medical records. However, such approaches are standard and necessary when working with large administrative data sets [27].

## Conclusions

In conclusion, we found that a simple modification of the original Liu comorbidity index provided reasonably comparable comorbidity ascertainment and allowed retention of a sizable and particularly vulnerable portion of chronic dialysis initiates. The benefits of this approach are a larger sample size, reduced healthier-survivor bias within the sample, and enhancement of the proportion of survival time available for outcome assessment. We conclude that nephrology health services researchers should consider using this modification for comorbidity adjustment if they are conducting observational studies with similar data sets and insurance eligibility requirements.

## Abbreviations

USRDS: United States Renal Data System; RCT: Randomized clinical trial; CMS: Medical Evidence Form (CMS 2728); CCI: Charlson Comorbidity Index; CMS 2728: Centers for Medicare & Medicaid Services Medical Evidence Form; ICD-9-CM: International Classification of Diseases, Ninth revision, Clinical Modification; MSE: Mean square error

## Competing interests

None to declare for each author.

## Authors’ contributions

SR participated in study conception and design, analysis planning, determination of inferences, and drafting of the manuscript. JW participated in study conception and design, analysis planning, determination of inferences, and manuscript preparation. JM conducted the statistical analysis, and participated in study design, analysis planning, determination of inferences and critical review of the manuscript. LD participated in statistical analysis and critical review of the manuscript. EE participated in study design, analysis planning, determination of inferences, and critical review of the manuscript. TS coordinated the research team, participated in study conception and design, analysis planning, determination of inferences, and critical review of the manuscript. All authors have read and approved the final manuscript.

## Authors’ information

SKR is Professor of Medicine and the Director of the Office of Scholarly, Academic & Research Mentoring (OSARM) in the Department of Medicine at the University of Kansas School of Medicine.

JBW is Associate Professor of Medicine at the University of Kansas School of Medicine and the Director of Inpatient Dialysis at the University of Kansas Hospital.

JDM is Associate Professor of Biostatistics at the University of Kansas School of Medicine and Director, Data Management and Statistics Core at the University of Kansas Alzheimer’s Disease Center.

LD is Senior Research Analyst in the Department of Biostatistics at the University of Kansas School of Medicine.

EFE is Professor of Medicine and The Sosland Family Endowed Chair in Preventive Medicine and Public Health at the University of Kansas School of Medicine.

TIS is Professor of Preventive Medicine and Public Health at the University of Kansas School of Medicine.

## Pre-publication history

The pre-publication history for this paper can be accessed here:

http://www.biomedcentral.com/1471-2369/14/51/prepub
